# Activation-Induced Cytidine Deaminase (AID)-Associated Multigene Signature to Assess Impact of AID in Etiology of Diseases with Inflammatory Component

**DOI:** 10.1371/journal.pone.0025611

**Published:** 2011-10-03

**Authors:** Diana Mechtcheriakova, Yury Sobanov, Gabriele Holtappels, Erika Bajna, Martin Svoboda, Markus Jaritz, Claus Bachert, Erika Jensen-Jarolim

**Affiliations:** 1 Department of Pathophysiology and Allergy Research, Center of Pathophysiology, Infectiology and Immunology, Medical University of Vienna, Vienna, Austria; 2 Department of Otorhinolaryngology, University Hospital Ghent, Ghent, Belgium; 3 Research Institute of Molecular Pathology, Vienna, Austria; MRC National Institute for Medical Research, United Kingdom

## Abstract

Activation-induced cytidine deaminase (AID) is expressed in B cells within germinal centers and is critically involved in class switch recombination and somatic hypermutation of immunoglobulin loci. Functionally active AID can additionally be detected within ectopic follicular structures developed at sites of chronic inflammation. Furthermore, AID may target non-Ig genes in B- and non-B-cell background. Therefore, AID-associated effects are of increasing interest in disease areas such as allergy, inflammation, autoimmunity, and cancer.

Pathway- or disease-relevant multigene signatures have attracted substantial attention for therapeutic target proposal, diagnostic tools, and monitoring of therapy response. To delineate the impact of AID in etiology of multifactorial diseases, we designed the AID-associated 25-gene signature. Chronic rhinosinusitis with nasal polyps was used as an inflammation-driven airway disease model; high levels of IgE have been previously shown to be present within polyp tissue. Expression levels of 16 genes were found to be modulated in polyps including AID, IgG and IgE mature transcripts which reflect AID activity; clustering algorithm revealed an AID-specific gene signature for the disease state with nasal polyp. Complementary, AID-positive ectopic lymphoid structures were detected within polyp tissues by *in situ* immunostaining. Our data demonstrate the class switch recombination and somatic hypermutation events likely taking place locally in the airways and in addition to the previously highlighted markers and/or targets as IL5 and IgE suggest novel candidate genes to be considered for treatment of nasal polyposis including among others IL13 and CD23. Thus, the algorithm presented herein including the multigene signature approach, analysis of co-regularities and creation of AID-associated functional network gives an integrated view of biological processes and might be further applied to assess role of altered AID expression in etiology of other diseases, in particular, aberrant immunity and cancer.

## Introduction

Adaptive immunity mechanisms ensure specificity for foreign antigens with virtually unlimited diversity during differentiation of T and B lymphocytes. In contrast to T cells, B lymphocytes have developed two additional independent steps to further diversify their receptors after antigen collision: somatic hypermutation (SHM) and class-switch recombination (CSR). Both SHM and CSR critically depend on the expression of activation-induced cytidine deaminase (AID) [1], [2]. AID is a member of the APOBEC family of cytidine deaminases, which acts via introduction of single-strand breaks into target DNA through deamination of cytosine for conversion to uracil. AID is currently considered as the only B-cell-specific factor required to trigger both SHM and CSR, when DNA breaks are specifically introduced into the variable or switch regions of Ig genes, respectively [3], [4]. In germinal centers (GCs) the AID expression is transient and is initiated in early centroblasts, is maximal in full-blown centroblasts, significantly decreases in centrocytes and is downregulated in plasma cells [5]. Additionally, AID-positive cells could be detected outside the GCs; a major fraction of this type of AID-positive cells resides within the subset of interfollicular large B lymphocytes [6], [7].

Clearly, such a potent mutagenic and recombinogenic enzyme needs to be tightly regulated at different levels to minimize the risk of unwanted DNA damage. A number of mechanisms restricting AID expression/activity to distinct cell types, time frames and target loci were identified [8]–[13]. Nevertheless, recent findings indicate that the presence of ectopic lymphoid structures can be detected in chronically inflamed tissues in several autoimmune disorders [14]; in synovium of rheumatoid arthritis the AID-positive follicular structures are directly implemented in promoting the production of pathogenic autoantibodies [15]. Local expression of AID and class switch recombination to IgE was shown in the bronchial mucosa of atopic and nonatopic patients with asthma [16] and within the oesophageal mucosa of patients with chronic oesophagitis [17].

Furthermore, breaches within the regulatory network seem to allow AID to target non-Ig genes within genomic DNA [18]–[21]. Thus, aberrantly expressed and/or aberrantly regulated AID may function as a general, genome-wide mutator [22] being involved in disease development of different etiology. AID as a node gene and the subsequent AID-associated events therefore receive increasing attention in disease areas such as inflammation, autoimmunity and cancer.

The phenotypic heterogeneity of human diseases presents a major challenge to advancing our in-depth understanding of disease mechanisms. However, there is good evidence to believe that establishment and validation of gene-, pathway-, or disease-relevant signatures provide tools for understanding the functional relevance of gene alterations in human diseases – not only for basic research but also for therapeutic target proposal, diagnostic tools, and monitoring of therapy response [23]–[26]. Different methods may be applied to address the role of a functional gene module in the etiology of a multifactorial disease at the level of gene alterations: (i) the *in silico* data-driven approach is based on the analysis of available microarray datasets and dissects gene-associated pathways into meaningful modules; the data analysis offer the advantage of a transcriptome-wide screening procedure but often lack the sensitivity for genes expressed at a low levels; (ii) a knowledge-driven approach uses a self-designed gene signature. In this case, a core set of interacting genes is assembled based on mining the scientific literature and/or with the help of bioinformatics, and is subsequently applied for the real-time PCR-based gene expression profiling. This methodology offers the detailed characterization of the input of one particular pathway while keeping limited amount of genes at the beginning of the study. Important advantage, on the other side, is the high sensitivity and reproducibility allowing quantitative profiling even of low-copy genes which are below the detection limits of microarray platforms. In the current study, we used the knowledge-driven approach to create an AID-associated 25-gene signature. This signature was evaluated in a disease model of benign, chronically inflamed tissue, namely in nasal polyposis. Chronic rhinosinusitis without nasal polyps, characterized by a modest inflammatory reaction, was used for immunopathological comparison as control tissue [27]. Nasal polyps are considered to be a model for persistent severe airway disease and do share the Th2-bias and the polyclonal IgE production with asthma, as recently shown [28]. Th2-cytokines and IgE also link nasal polyps to comorbid asthma, as demonstrated by the analysis of factors within the polyp tissue, which are associated with asthma [29]. The immunopathological mechanisms underlying the nasal polyp formation are not completely resolved. Whereas the focus of investigations was on T-cells and their subpopulations [30]–[32], the impact of B-cells has scarcely been analysed. B lymphocyte infiltrations – diffuse accumulations and those organized to follicles [33], B cell attractant chemokines [34] and high levels of IgE type antibodies were shown to be present within polyp tissue [35], [36]. Thus, expression of functional AID within nasal polyp tissue has been presumed but never conclusively demonstrated. The approach presented herein allows assessing the role of AID-associated events in the molecular mechanisms underlying the initiation and/or progression of an inflammation-driven disease.

## Methods

### Profile of study patients

A panel of 33 specimens of nasal mucosal tissues from patients with chronic rhinosinusitis without nasal polyps (CRS; n = 15) and with nasal polyps (NP; n = 18) was obtained from the Department of Oto-Rhino-Laryngology of the University Hospital of Ghent, Belgium. All samples were obtained during routine endonasal sinus surgery in consecutive patients scheduled for surgery unrelated to the study. The diagnosis of sinus disease was based on history, clinical examination, nasal endoscopy and CT-scan of the sinuses according to the EP3OS guidelines [37] None of the subjects used oral or nasal corticosteroids four weeks before surgery or antibiotics within the last two weeks. All patients provided written informed consent, and the ethics committee of the Ghent University Hospital approved the study. Clinical parameters including history of atopy, asthma, aspirin hypersensitivity (ASA), total IgE antibody levels, IgE specific to Staphylococcus aureus enterotoxins (SAE-IgE), levels of eosinophil cationic protein (ECP) and IL5 in tissue homogenates are summarized in **Table S1**. Samples processing and measurement procedures were performed as described [33]. Each sample was separated to proceed with protein and total RNA isolation as described [33].

### Primer design, real-time PCR analysis, data visualization, pathway analysis, statistics

Gene expression profiling was performed by real-time PCR on ABI PRISM 7900HT (Applied Biosystems) in 96-well plates using the SYBR Green detection system as described [38]. Primers were designed using “Primer Express 2.0” software and validated using a normal tissue panel (Takara, Clontech). Algorithm for detection of IgM, IgG, and IgE mature transcripts and results of BLAST analysis of IgG forward and reverse primers are available as **Table S2**. Sequence for CD23b was reconstructed based on the comparison of virtually translated nucleotide sequence of intron 2 of CD23 gene and previously published N-terminal amino acid sequence of CD23b variant [39]. For relative quantification, data were analyzed by ΔΔCT method using SDS 2.3. (Applied Biosystems) and normalized to the average of housekeeping genes (HKG) as elongation factor (EF1A), beta 2 microglobulin (b2M), actin b (ACTB), and ubiquitin C (UBC). For the absolute gene quantification, plasmid overexpressing gene of interest was used as external standard [40], [41]. Plasmids expressing the full-length AID (pUHD10S-Flag-AID-fl) and the naturally occurring splice variant with the truncated C-terminus (pUHD10S-Flag-AID-dE4) were kindly provided by Prof. Xiaosheng Wu [13]; plasmid encoding full-length human ACTB was from Invitrogen. Cluster 3.0 and TreeView programs (http://bonsai.hgc.jp/~mdehoon/software/cluster/manual/index.html) were used for computational analysis and graphical representation of datasets. The data-driven, AID-associated gene network was created using the Ingenuity Pathway Analysis Software (http://www.ingenuity.com). Statistical analyses were performed using GraphPad Prism 5.0 and SYSTAT 12 programs. Differences between diseased groups and alignments with clinical parameters were analyzed using log2 values of variances. For Student t tests, 2-way analysis of variance, *p* values of 0.05 or less were considered significant.

### AID-associated 25-gene signature

Based on the knowledge-driven approach of B cell biology and NP data mining, a signature of AID-associated genes was assembled. The 25-gene “AID signature” includes the full-length AID-FL and the alternative AID splice variant AID-Δex4; activators and suppressors in AID regulation such as PAX5, IRF8, ID2, ID3, EGR1/2/3 [42]–[44]; immune cell markers as CD19, CD3, CD14, CD86, CD21L [15], [45]; Th2 cytokines as IL5, IL13 (IL4 was excluded based on the trace levels of expression in both diseased groups); low and high affinity IgE receptors as CD23 (variant a and b), FceRI alpha, beta and gamma subunits; IgM, IgG, IgE mature transcripts. Tables with NCBI accession numbers, gene symbols and synonyms, primer sequences and short functional gene descriptions are available as supplementary files (**Table S3**, **Table S4**). In summary, the composition of the signature created around AID as a node gene allows to assess AID expression and AID activity as proven by the class switch recombination-based formation of IgG and IgE mature transcripts; the presence of tissue infiltrating immune cells such as B cells, T cells, monocytes, and follicular dendritic cells being indicative for various stages of lymphoid organization; the expression pattern of low- and high-affinity IgE receptors mediating numerous IgE-related immune responses; and Th2 polarization.

### Immunostaining on paraffin-embedded tissue sections and AID-positive Raji cell line

Raji, lymphoblastoid cells derived from a Burkitt lymphoma, were obtained from ATCC (Manassas, VA). Cytospins of Raji cells were fixed with methanol/acetone solution (1∶1 v∶v) for 12 min at −20 C°. Immunofluorescence with Raji cells was generally performed as described [38]. Paraffin blocks of disease specimens were prepared using the complete isolated tissue; these disease specimens do not overlap with patient population taken for gene expression profiling. Paraffin-embedded 5 µm-thick sections underwent routine staining with haematoxilin and eosin. Sections of NP specimens with lymphoid aggregates displaying a radial cell number greater than 10 cells (n = 5) and sections of CRS patients (n = 4) were taken for CD20 and AID staining. To detect AID, two different antibodies were used: clone ZA001 mouse IgG1-kappa (Invitrogen) and clone EK2-5G9 rat IgG2b (Cell Signaling). ; anti-AID antibodies were previously shown to be functional in immunostaining of cells transfected with the plasmid encoding AID [12] and of AID-positive cells in paraffin-embedded tissue sections [46], [47]. For immunohistochemistry method, DAKO EnVision+, Peroxidase system (DAKO, Glostrup, Denmark) was used. Sections were counterstained with haematoxylin for nuclear visualization. Alternatively, a fluorescent staining with biotinylated rabbit anti-rat Ig or anti-mouse Ig secondary antibodies (Invitrogen, Paisley, UK) followed by streptavidin conjugated to Alexa dyes was used (Invitrogen, Paisley, UK). Nuclear counterstaining was performed with DAPI (Roche, Mannheim, Germany). In our experimental settings, both anti-AID antibodies showed identical results (data not shown); immunohistochemistry method was preferable to immunofluorescence for AID staining within NP tissues. CD138 (clone BA38, from AbD Seroteck, Oxford, UK) was used as a marker for plasma cells [48]; CD20 (clone L26, from Thermo Scientific, Cheshire WA7 1PR, UK) was used as general B cell marker. Polyclonal Rabbit Anti-Human IgE was from DAKO (Glostrup, Denmark). TissueFAXS (TissueGnostics, Vienna, Austria), a fully automated multi-channel immunofluorescence tissue analysis system was used for the acquisition of diseased specimen/paraffin-embedded tissues as well as of Raji cells. For acquisition the 20x/0.5 or the 40x/1.3-oil objectives were used (EC Plan_NeoFluar, Zeiss). Filter sets were from Chroma TechnologyCorp (DAPI 350/460 nm; FITC/Cy2 470/525 nm; TxRed/Cy5 620/700 nm). Images were processed using Adobe Photoshop CS2 software.

## Results

### Expression profiling of functional AID in specimens from patients with chronic rhinosinusitis without or with nasal polyps

**Figure 1:**
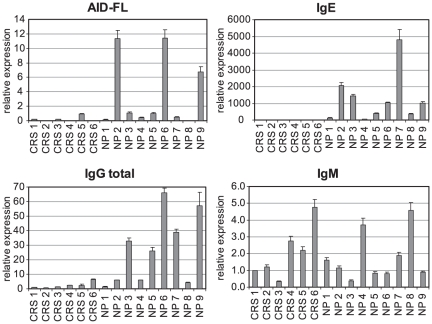
Expression profiles of AID and Ig transcripts in individual samples of patients with chronic rhinosinusitis without (CRS) or with nasal polyps (NP) assayed by real-time PCR. Expression profiles were analyzed using the ΔΔCT method for relative quantification. Expression levels of full-length AID (AID-FL), mature transcripts of IgM, IgG total, and IgE mRNAs were normalized to the average of HKGs and shown relative to CRS1. Results are shown as mean values in one experiment ± SD and are representative of two independent runs.

We designed primer pairs to detect AID mRNA of human origin and performed expression profiling in normal tissues (multiple tissue panels, Clontech) (Text S1 and **Figure S1**). Furthermore, we established a real-time PCR-based approach to detect IgM, IgG (total), and IgE mature transcripts within the same specimen. This highly sensitive and quantitative method can be routinely used for detection of Ig transcripts within the tissues from large cohort of samples. Detection of IgG or IgE mature transcripts was used to assess AID activity as the ability to produce different isotypes of antibodies is a functional consequence of the active AID molecule. We next analyzed the AID expression pattern in specimens from patients with chronic rhinosinusitis without nasal polyps (CRS) in comparison to the one with nasal polyps (NP). Within the pilot experiment, six CRS samples and nine NP samples were profiled. As shown on **Figure 1**, CRS samples were characterized by non-detectable or trace AID mRNA levels, whereas strongly elevated expression levels of AID indicative for initiation of class switch recombination were detected in 3 out of 9 NP samples. Marked inter-patient variability of AID mRNA levels within the NP group might be explained by the transient nature of AID expression and/or various stages of B cell response for each individual. IgG total mRNA levels were significantly increased in the NP group in comparison to the CRS patients (p<0.01). IgE were detected at trace amounts in CRS, while being strongly elevated in the NP group (p<0.001). In contrast, IgM mature transcripts were detected in both CRS and NP groups characterizing the presence of B cells in both diseased groups. Of note, strongly enhanced AID expression detected in NP2, NP6, and NP9 was associated with elevated expression of either IgE and/or IgG mature transcripts. Furthermore, subpopulation within the NP group, characterized by the moderate AID expression, showed increased IgE and/or IgG expression levels. In line, absence of AID expression in the CRS group was accompanied by non-detectable or low IgE and IgG transcription, while levels of AID-independent IgM transcription were comparable to those of the NP group. Partially overlapping expression patterns of AID and IgG/IgE mature transcripts within the NP group might be explained by the transient nature of both AID and IgG/IgE transcription and the time-shift in kinetics between these two events - the preceding AID expression followed by the AID-mediated CSR resulting in the production of Ig isotype switched mature transcripts [49]. Of particular importance, a strong positive correlation (r = 0.747; p<0.001) was found between IgE mature transcript levels estimated by real-time PCR technique and total IgE protein levels determined in supernatants after tissue homogenization (**Table S1**). These data suggest (i) the AID-positive B cells to be present within NP tissue; (ii) at least part of detectable IgE protein within NP is based on AID-driven CSR.

### Comparative profiling of AID-FL and the splice variant

Alternative splicing may be a potential mechanism that controls AID action under physiological conditions. The naturally occurring splice variant lacking exon 4, AID-Δex4 (AY536517), encodes a C-terminally truncated product, which is characterized by the complete lack of CSR activity, while showing hyper-SHM activity [13]. Herein, we compared mRNA levels of the full-length AID transcript, AID-FL, and of the splice variant AID-Δex4 within the same tissue. The panel under investigation was composed from normal human tissues which were found to be AID-positive on the mRNA level (data are available as Figure S1 and detailed in Text S1) and NP specimens; lymph node (total RNA, Ambion) was included as a positive control. The ratio between AID-FL and AID-Δex4 was characterized by a strong inter-tissue variability (**Table 1**). Thus, in salivary gland, thymus, thyroid gland, lymph node, NP2, and NP6 the AID-FL mRNA levels exceeded those of the AID-Δex4 variant, while in the tissues with moderate levels of total AID, mRNA expression of AID-Δex4 was comparable to or higher than the full-length transcript (e.g. NP4, NP5). The results indicate occurrence of alternative splicing *in vivo*.

**Table 1:** Comparative analysis of AID-FL and AID-Δex4 mRNA levels.

tissue	AID-FL	AID-Δex4	ratio
			AID-FL∶AID-Δex4
bone marrow	12.19±3.3	8.16±3.1	1.49
kidney	15.46±3.1	39.46±3.4	0.39
salivary gland	180.85±41.9	53.28±4.6	3.39
thymus	539.11±57.2	62.56±8.2	8.62
thyroid gland	168.17±43.9	50.65±11.2	3.32
lymph node	1658.69±93.3	209.39±11.9	7.92
NP2	284.85±65.6	60.33±13.4	4.72
NP3	40.93±12.0	35.13±6.4	1.17
NP4	13.58±4.4	38.67±11.0	0.35
NP5	44.41±13.5	75.5±19.6	0.59
NP6	264.70±37.9	41.98±11.7	6.31

An absolute quantification was performed using recombinant DNA-based external standards (Standard Curve Assay Getting Started Guide, Applied Biosystems). Efficiency and linearity of the standard curve (*R* ^2^) for AID-FL, AID-Δex4, and ACTB were *E*_AID-FL_ = 1.99, *R* ^2^_AID-FL_ = 0.999; *E*_AID-Δex4_ = 2.03, *R* ^2^_AID-Δex4_ = 0.998; *E*_AID-FL_ = 1.99, *R* ^2^_AID-FL_ = 0.999; *E*_Actin_ = 2.01, *R* ^2^_Actin_ = 0.999. Results are expressed as absolute cDNA copy number of target gene per 10^6^ copy numbers of ACTB as HKG. Shown are mean values from three independent experiments each performed in duplicate ± SEM.

### AID-positive ectopic lymphoid structures within nasal polyp tissues assayed by *in situ* immunostaining

**Figure 2:**
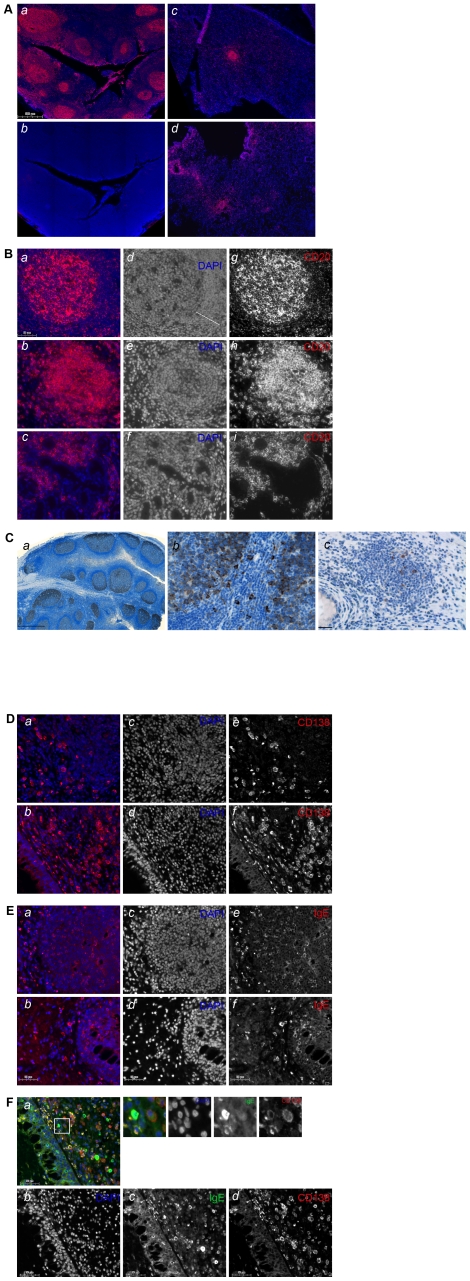
AID expression in nasal polyp tissues assessed by in situ immunostaining. (A) CD20 staining in fully developed GCs in tonsil tissue (CD20, a; isotype control, b) and two representative NP specimens (c, d); scale bar: 500 µm. (B) CD20 staining: higher power views of GC in tonsil (a) and of two types of B cell infiltrates in NP as prominent follicles (b) and accumulations around the glandular structures (c) are shown. In addition to the merged images (a, b, c; red channel for CD20 and blue channel for DAPI), pictures of individual channels are included (d, e, f for DAPI; g, h, i for CD20); the presence of the mantel zone in fully established GC within tonsil tissues is indicated by a white dotted line. Scale bar: 50 µm. In Figure 2, the individual channels are shown in black/white, whereas merged images are shown in color. (C) AID-positive GCs in tonsil tissue (a, the low-power view; b, the higher-power view) and follicular structures within NP with AID-positive cells (c) are shown; Scale bar for a is 1000 µm; scale bar for b, c is 50 µm. (D) Two areas representative for accumulation of CD138-positive cells within NP tissues are shown: around follicular structures (merged, a; DAPI, c; CD138,e) and in the close proximity to the boarder of NP body (merged, b; DAPI, d; CD138, f). Scale bar: 50 µm. (E) Representative pictures of IgE-positive cells detected within follicular structures and their surrounding cell populations and within B cell aggregates around the glandular structures (merged a, b; DAPI, c, d; IgE, e, f). Scale bar: 50 µm. (F) Double staining of IgE (green) and CD138 (red) within NP (merged a; DAPI, b; IgE, c; CD138, d); insert: example of double positive B cell is shown. Scale bar: 50 µm.

Enhanced AID expression in NP tissues detected by real-time PCR may be based on increased migration of activated B cells and/or ectopic expression. Therefore, to estimate ectopic lymphoid structure formation and presence of AID-positive cells, *in situ* immunostaining analysis of NP specimens was performed. Tonsils (a secondary lymphoid organ with fully organized functional GC structures) and the Raji cell line were used as reference tissue/cell type for AID expression. As expected, immunofluorescent staining of tonsils for CD20 revealed strong expression in the GCs of lymphoid follicles (**Figure 2, A**, *a*). CD20 immunostaining analysis of NP specimens revealed that lymphocytic infiltrates are able to evolve into follicular structures (**Figure 2, A**, *c*, *d*); the data are in line with previously published observations [33]. Lymphoid follicles developed within NP tissues differed from those in tonsils in morphology; thus, the mantel zone characteristic for active GCs within tonsils (**Figure 2, B**, merged, *a*; DAPI, *d*; CD20, *g*) was not fully established in NP-derived follicular structures (**Figure 2, B**, merged, *b*; DAPI, *e*; CD20, *h*) likely suggesting their transient nature. Different patterns of B cell accumulations were found to coexist within the same specimen; in addition to CD20-positive follicles, B cell aggregates around the glandular structures, the subepithelial mucosal glands contributing to the secretion of mucus, composed of more than fifty CD20-positive cells could be detected (**Figure 2, B**, merged, *c*; DAPI, *f*; CD20, *i*).

To visualize the AID protein expression, immunofluorescent or immunohistochemical staining protocols were established as demonstrated for Raji cells on **Figure S2**; however, immunohistochemistry was superior to AID staining for NP tissues. Immunohistochemical staining of tonsils revealed strong AID expression in the GC; in fully developed GC, AID staining was predominantly seen in the dark zone (**Figure 2, C**, *a*). Consistent with a previous report [6], extrafollicular AID-positive cells were as well readily detected (**Figure 2, C**, *b*). Within NP tissues, AID-positive B cells were predominantly localized within the follicular structures (**Figure 2, C**, *c*). Of note, CD20-positive lymphocytic aggregates around the glandular structures, which were not organized into follicles, were found to be negative for AID expression (Figure S3). In contrast to NP tissues, no CD20-positive follicular structures were detected within CRS specimens; similarly to NP, lymphocytic non-follicular aggregates were AID-negative (data not shown).

In addition, within NP tissues the CD138-positive plasma cells were found to be localized outside the follicular structures (**Figure 2, D**), which is in line with the knowledge that plasma cells do not express the CD20 marker [6]. Additional areas showing accumulation of CD138-positive cells were found in the close proximity to the boarder of the polyp tissue body (**Figure 2, D**) and to the glandular structures. IgE-positive cells of different intensities were readily detected within the same areas as CD138-positive cells and in addition within follicular structures (**Figure 2, E**). Double staining for IgE and CD138 confirmed the presence of an IgE-positive B-cell subset (**Figure 2, F**); single stained IgE-positive cells might be attributed to an effector cell population based on the bi-lobed form of the nuclei (e.g. eosinophils, basophils; Figure S4).

In summary, detection of AID and mature IgE transcripts by real-time PCR analysis accompanied by immunostaining data suggest that the nasal polyp tissue might contain functionally active, AID-positive ectopic follicular structures. Thus, the data indicate that nasal polyposis is a relevant disease model to study AID-associated responses with a presence of ongoing class switch recombination in benign, chronically inflamed tissues.

### AID-associated gene expression signature: linking gene expression data with a functional network

**Figure 3:**
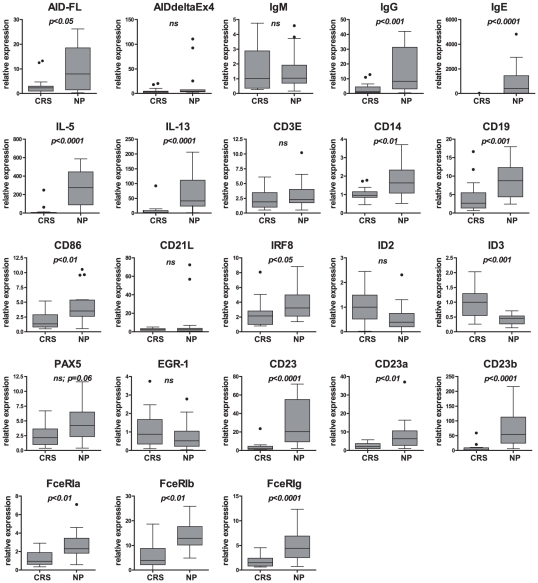
Differential expression of AID-associated genes in specimens with nasal polyps (NP) in comparison to the one without nasal polyps (CRS). (A) Box-plot analysis of gene expression profiling dataset of CRS (n = 15) and NP (n = 18) specimens. The box represents the distribution of values; a line across the box represents the median; the box stretches from the lower hinge (the 25th percentile) to the upper hinge (the 75th percentile). p value is shown for each gene (t test for log2 values of variances). All modulated genes with the significant difference between two diseased groups, including AID, passed the Holm-Bonferroni correction for multiple testing.

A panel of 33 clinically characterized samples with chronic rhinosinusitis without nasal polyps (CRS; n = 15) and with nasal polyps (NP; n = 18) was profiled with primer sets from the 25-gene “AID signature” specified in Methods and **Tables S2–S4**. Genes significantly affected in NP in comparison to CRS (*p*<0.05 – *p*<0.0001) were identified: upregulated AID, IL13, IL5, CD23 total and CD23a, CD23b isoforms, FceRIa, FceRIb, and FceRIg subunits, IgG and IgE mature transcripts, CD14, CD19, CD86, PAX5 (tendency; *p* = 0.06), IRF8; downregulated ID3 (**Figure 3**). Next, we performed an alignment of expression datasets with the patient's clinical parameters (summarized in **Table S1**) such as sex, history of atopy, asthma, and aspirin hypersensitivity (ASA). Data analysis revealed significant associations between (i) atopy and CD23/CD23b, IL13, IL5, CD19, CD86, CD14, IgG, IgE, FceRIa, FceRIg, and ID3; (ii) asthma and CD23/CD23a/CD23b, IL13, IL5, CD19, CD14, IgG, FceRIg, AID (tendency, *p* = 0.051), and IgE (tendency, *p* = 0.052) (**Table S5**). No associations with sex or ASA were found.

**Figure 4:**
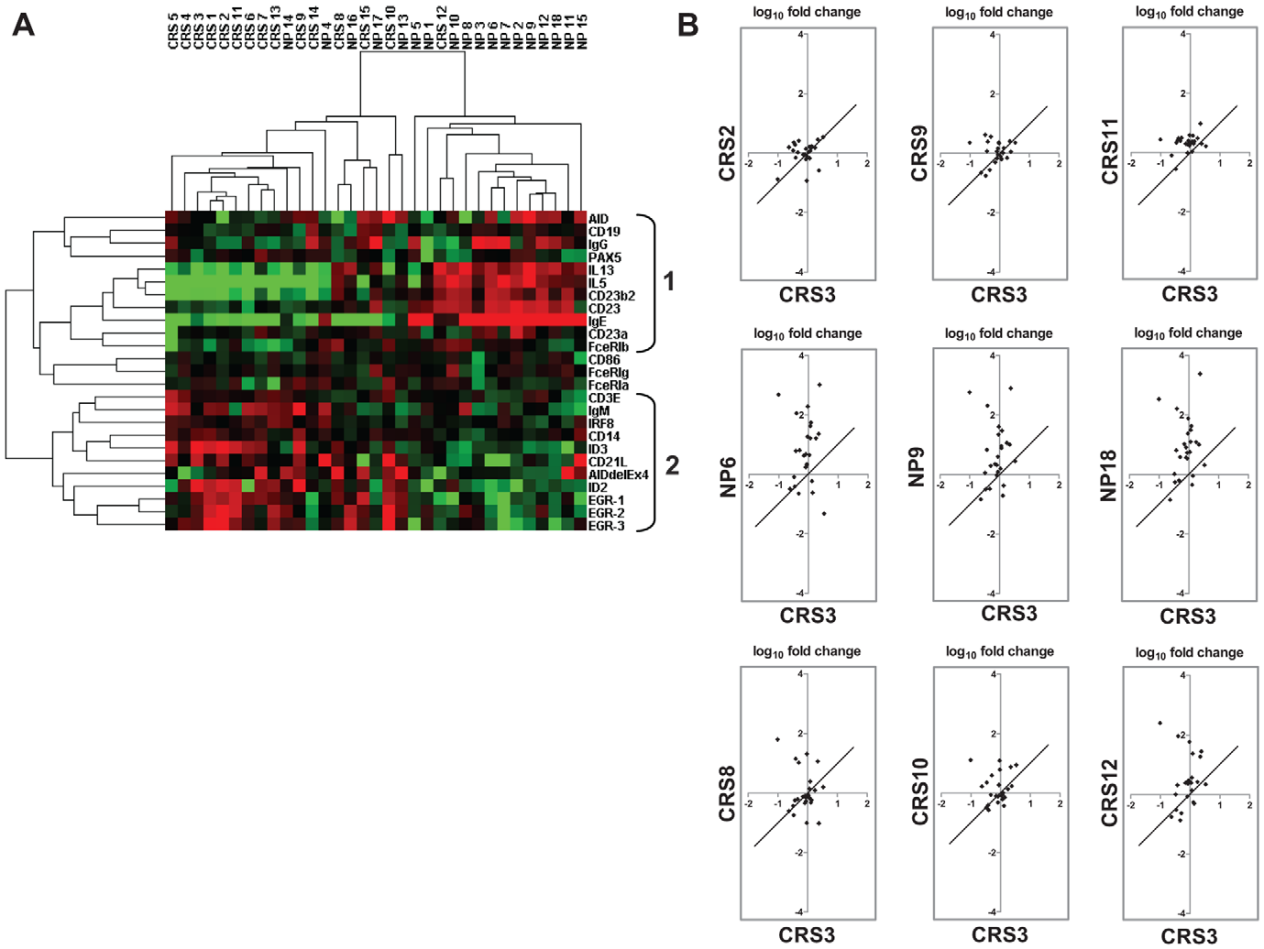
Hierarchical clustering of expression datasets and scatter plots of patient-orientated profile. (A) The subset of 25 genes was analyzed against a dataset derived from the real-time PCR-based profiling of a panel of 33 specimens from patients with chronic rhinosinusitis with nasal polyps (NP; n = 18) in comparison to the one without nasal polyps (CRS; n = 15); Pearson uncentered hierarchical clustering was applied. Shown is a result of a clustering run representing a pair of trees, one for genes and one for arrays/samples (Cluster/TreeView programs). Sub-signatures 1 and 2 orchestrated around AID and IDs/AID-Δex4 (AIDdelEx4 here), respectively, are indicated. Color code: red indicates induction and green - repression; according to Cluster/TreeView [50], color hue does not reflect the exact magnitude of gene alteration. (B) log transformation plots represent the patient-orientated gene expression “fingerprint”; the figure depicts the relative expression level of each gene between CRS or NP specimens (y-axis) and CRS3 specimen (x-axis); axis – log scale. Representative scatter plots of three patients from the CRS group (CRS2, CRS9, CRS11) and three patients from the NP group (NP6, NP9, NP18) are shown. These specimens were selected from two main clusters, separating CRS and NP samples (Figure 4, A). Scatter plots of three CRS specimens (CRS8, CRS10, CRS12) from a mixed population are shown in addition.

Hierarchical cluster analysis was used for further data interpretation and visualization. This algorithm arranges the data into a tree structure providing information about the relationship between the samples and between the genes [50]. The samples fall into two main clusters, separating majorities of CRS and NP samples with only few samples forming a mixed population (**Figure 4, A**). Furthermore, two gene sub-signatures emerged. Sub-signature 1 can be designated as AID-associated (n = 11 genes). Within this sub-signature, the AID-positive cluster contained a higher number of NP samples, while the AID-negative cluster contained the majority of CRS specimens. Hierarchical clustering revealed close associations between subset of genes IL13–IL5–CD23b–CD23–IgE–CD23a–FceRIb, which are further linked to AID–CD19–IgG–PAX5. Estimated coefficients of correlation were found to be >0.6, *p*<0.0001 for IL5–CD23/CD23b, IL13–IL5, IL13–IgE, IL13–CD23/CD23a/CD23b (for all genes, Pearson correlation matrix and matrix of probabilities, matrix of Bonferroni corrected probabilities are available as **Table S6**). Of importance, AID was found to have a positive correlation (correlation coefficient>0.6, *p*<0.0001) with IL13, IL5, CD19, CD23, CD23a, and PAX5. AID showed less stringent positive correlation with IgG mature transcripts (correlation coefficient = 0.471, *p* = 0.005) and IgE (correlation coefficient = 0.417, *p* = 0.014). Partially overlapping time-courses and transient nature of both AID and IgG/IgE transcription might explain these results [42]. No significant correlation was found between AID expression and expression pattern of CD21L, an isoform specifically expressed by follicular dendritic cells [15], [45]. No significant correlation was revealed between AID full-length transcript and AID-Δex4. Sub-signature 2 (n = 11) was formed around the transcriptional repressors ID2/ID3; splice variant AID-Δex4 was included in this gene cluster. Inverse to the sub-signature 1, higher expression values were characteristic for CRS specimens and the CRS/NP mixed population, while the NP group presented with lower values.

### Patient-orientated gene expression pattern

Arrangement of datasets for each specimen across all genes is able to provide the gene expression pattern characteristic for each individual sample and therefore being patient-orientated. Scatter plots of three patients from the CRS group (CRS2, CRS9, CRS11) and three patients from the NP group (NP6, NP9, NP18) are shown on **Figure 4, B**. The patients were selected from two main sample clusters separating CRS and NP groups and therefore are representative for each group. As expected, the disease-group-characteristic gene distribution patterns are evident; however, the patient-specific differences within gene expression signature are apparent as well. Of importance, scatter plots of CRS specimens from the clustering-based mixed population (CRS8, CRS10 and CRS12) differed from both CRS-specific and NP-specific patterns showing rather the intermediate stage.

## Discussion

Disease-relevant multigene signatures have attracted substantial attention for therapeutic target proposal, diagnostic tools, and monitoring of therapy response [23], [24], [26]. Development of diagnostic and/or prognostic signatures considered to be an important step towards personalized medicine. To assess the role of B cell-driven responses associated with altered AID expression, we used a self-created gene signature to perform a comparative analysis of the transcriptional programs characteristic for chronic rhinosinusitis with or without nasal polyps. The composition of the 25-gene signature allows to estimate (i) the presence of tissue infiltrating immune cells such as B cells, T cells, monocytes, and follicular dendritic cells, (ii) AID expression (the full length and AID-Δex4), (iii) AID activity proven by the class switch recombination-based formation of IgG and IgE mature transcripts, (iv) various stages of lymphoid organization, (v) the expression pattern of low- and high-affinity IgE receptors mediating numerous IgE-related immune responses. This core set of genes, which is of particular relevance for inflammatory and allergic disorders, may be further extended with additional candidate genes using bioinformatics approaches to fulfil the criteria for other disorders.

**Figure 5:**
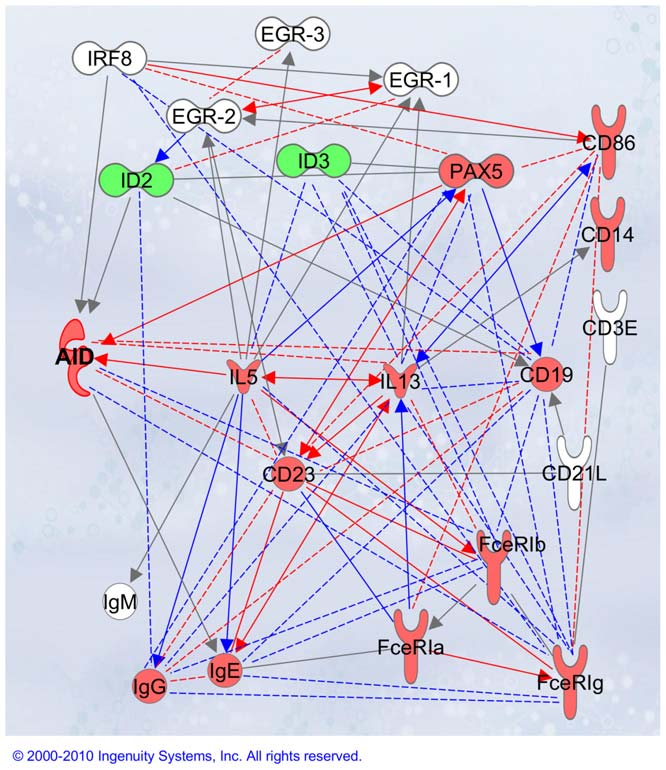
AID-associated gene network. Multigene signature-based network displaying AID as a key gene was created using the Ingenuity Pathway Analysis Software (IPA; http://www.ingenuity.com). The color code of transcripts indicates up- (red) and down-regulation (green) of genes in the NP disease group in comparison to rhinosinusitis without polyps. Solid lines in grey display the IPA-identified direct associations between transcripts; solid lines in red or in blue reflect the IPA-identified associations overlapping with the 25-gene-identified molecular interactions. The multigene approach including the analysis of co-regularities can be extended to find not-yet-identified functional links between the genes under investigation; statistically significant, study-based biological associations (SYSTAT program; Table S6) are displayed by dashed lines; color code: red for correlation coefficient ≥0.6, p<0.0001; blue for correlation coefficient <0.6, p<0.05.

Chronic rhinosinusitis was used as an inflammation-driven disease model. Disease development of chronic rhinosinusitis might take different directions; despite ongoing progress in research [27], [29], [32], [51], [52], it is yet unclear what mechanisms drive the disease towards the nasal polyp formation. In this study we provide, to our knowledge, the first demonstration that AID (the full-length transcript and the splice variant) is expressed within the nasal polyp tissue. Profiling of B cell markers (e.g. IgM transcripts, PAX5, CD23a, CD19) is indicative for the presence of infiltrating B cells in tissues of both diseased groups; yet comparison of gene expression patterns using a 25-gene signature confirmed an AID-associated signature for the disease state with NP (summary **Figure 5**). AID indicates initiation of CSR. Mature IgE transcripts, resulting from DNA excision and ligation of the V(D)J sequences to the constant epsilon region, indicate the presence of IgE-switched B cells. The expression profiling data are supported by immunostaining which demonstrate the existence of AID-positive ectopic follicular structures and IgE-switched B cells within nasal polyp tissue, suggesting class switch recombination and somatic hypermutation events to take place locally in the airways – at least for the sub-group within NP population. Complementary to local maturation and priming for class switching, increased migration of activated B cells into NP tissue and accumulation of long-lived plasma cells producing high levels of IgE antibodies cannot be excluded as an additional mechanism as suggested in recent study of Patadja et al [34]. The biological responses initiated within ectopic follicular structures may have various regulatory functions influencing the disease pathogenesis and/or disease resolution. Given the focus of the current study to the multigene signature approach, an open question remains whether a number of AID-positive follicles developed within polyp tissue correlate with clinical parameters.

Here, we report the results of comparative profiling of the AID-FL and the splice variant AID-Δex4 encoding a C-terminally truncated product; the latter was demonstrated to have strictly different enzymatic activity compared to the full-length transcript [13]. Furthermore, the C-terminus contains a determinant for AID cytoplasmic retention, which hampers diffusion to the nucleus, competes with nuclear import and is crucial for maintaining the predominantly cytoplasmic localization of AID [12]. Therefore, the analysis and quantification of AID-Δex4 mRNA, predicted and confirmed ([53] and DM, unpublished) to give rise to a C-terminally truncated AID protein with increased resident time in the nucleus, is of particular interest. It is essential to note that both AID-FL and AID-Δex4 mRNAs can be detected in NP specimens and in some normal non-lymphatic tissues as well (**Table 1**). One example of AID-positive tissue is thymus. The fact of AID expression in the thymic medulla at the mRNA as well as functional protein level was noted previously [7]; however, the biological relevance of AID expression within a B cell subset in the thymus of adults under physiological conditions is still poorly understood. For the majority of tissues under investigation the AID-FL mRNA levels exceeded those of the AID-Δex4 variant; keeping in mind constant nuclear localization of AID-Δex4 protein transcript, even low expression levels might have significant functional consequences. The method applied in the current study does not allow to determine whether AID-FL and AID-Δex4 are both expressed within the same cell or in different cells in a “one variant per cell” pattern as discussed recently by Wu et al [13]. During normal, physiological responses the sequential expression of AID variants may occur. Initial B-cell activation may trigger full-length AID expression, which drives predominantly the Ig class switch recombination processes; while at a later phase, the alternative splicing occurs and the AID-Δex4 truncated variant, characterized by a strong hyper-mutational potential, additionally modulates the affinity of the available antibody repertoire. The AID-Δex4 truncated transcript may be stored in memory and/or plasma cells; preferential expression of AID-Δex4 in plasma cells was indeed demonstrated [13]. Despite we did not find any specific expression pattern of AID-Δex4 in nasal polyps, the established algorithm can be further applied to test the hypothesis whether the ratio [AID-FL∶ AID-Δex4] might have a prognostic value for certain diseased conditions including chronic inflammation, autoimmunity, and cancer and/or might correlate with an increased potential for AID off-target effects.

It is of importance, that the multigene signature approach applied in the current study allows to differentiate the state of development/organization of lymphoid structures occurring at sites of chronic inflammation and/or recurrent antigenic stimulation; this is based on the analysis of associations between AID and the follicular dendritic cell marker CD21L [15], [45]. Thus, the study of Humby and colleagues [15] provided strong evidence that ectopic lymphoid structures formed within the synovial membrane of patients with rheumatoid arthritis were characterized by the presence of both AID-positive B cells and CD21L-positive follicular dendritic cells; moreover, a significant positive correlation was found between expression patterns of AID and CD21L mRNA. Therefore, these lymphoid aggregates were attributed to follicular structures resembling GCs. In the case of NP tissues, we could not detect significantly increased CD21L mRNA levels in patients with polyps; furthermore, no correlation was found between AID and CD21L expression patterns. Thus, organization and possibly functionality of lymphoid aggregates seem to be different to those described in chronically inflamed tissues in patients with rheumatoid arthritis. Follicular structures within NP tissue were found to be functionally active but not entirely organized as GCs. The data are in agreement with ongoing discussions that the forces driving B cells to form unique classes of active follicular structures with specialized functions are diverse and robust [54].

PAX5 - the unique transcription factor of B cell identity, which is necessary and sufficient for B cell development from the pro-B cell to the mature B cell stage [55]–[57] was detected in both diseased groups, however, with tendency for enhanced expression in NP. PAX5 functions both as a transcriptional repressor and as an activator on different target genes. Thus, among others, it represses Notch1 [57] and FLT3 [58] and it activates CD19 [59], BLNK [60], and AID [61]. Furthermore, the unexpected close correlation between AID and CD23 shown in the present study suggests a common transcriptional network in the regulation of these genes. Given that CD23 and particularly CD23a, showed a positive correlation to PAX5, one would predict that PAX5 is a candidate transcription factor involved in the upregulation of both AID and CD23 in B cells. Indeed, functional PAX5 binding site(s) were previously identified within the CD23a promoter [62].

Thus, some of the identified regularities in gene distribution patterns have already been proven by other groups in cell-based assays. Therefore, the multigene approach presented herein including the analysis of co-regularities can be further extended to find not-yet-identified functional links between the genes under investigation (**Figure 5**). Of interest, expression of FceRIg mRNA (signaling subunit of the high affinity IgE receptor) was upregulated in the NP group. Furthermore, the expression pattern of FceRIg was found to correlate positively with those of CD14, CD86, FceRIa as well as with AID, CD19, CD23, IgE and IgG transcripts, IRF8 and PAX5. Correlation with B cell identity markers suggests that FceRIg transcription could be triggered in B cells at a certain stage of their activation. Of note, FceRIa – the functional extracellular subunit of the FceRI receptor complex – did not reveal the list of regularities described above. Thus, the FceRI receptor complex might be regulated via the signaling gamma chain whereas the alpha chain is constitutively “ready”. Alternatively, the FceRIg chain might build up a signaling complex with other activating receptors besides FceRIa. Indeed, such examples were previously described for other cell types: when expressed by plasmacytoid dendritic cells, FceRIg was found to cooperate with BDCA2 [63]; on a subset of Treg cells, FceRIg chain functions within the TCR complex [64]. For B cells, a functional cooperation partner for FceRIg besides FceRIa has not yet been demonstrated to our knowledge.

Remarkable data suggest that EGR (early growth response) family members might also be involved in AID-associated biological responses [42], [65] including the fact that Egr-1 null mice showed increased levels of serum IgE [43]. Thus, according to our working hypothesis this transcription factor may be a regulatory player in the AID-associated network. However, expression profiling revealed only a tendency for reduced expression of EGR-1 mRNA in NP in comparison to the levels in rhinosinusitis without polyps, possibly “masked” by those cell types in NP tissue with steady or increased EGR-1 expression. Nevertheless, clustering and correlation analysis revealed clear associations of EGR family members with the transcriptional repressors ID2/ID3 jointly forming the AID-Δex4-included sub-signature 2. Thus, our data suggest a repressor function of the EGR-1 transcription factor in functionally active follicular structures.

IL5 was included as a positive control gene in the current study. The specific knowledge on the role of IL5 in CRS with NP as discovered previously [66] and the possibility of IL5 antagonism *in vitro* [67] led to the clinical application of humanized anti-IL5 antibodies for the treatment of nasal polyposis [68], [69]. However, only a subgroup of patients responded to this treatment pointing to the heterogeneity of the disease etiology on the one side, and of the patient population on the other side. Additionally, the data indicated that further target gene(s) might be considered for treatment of disease in single therapy or combined therapy with anti-IL5 agents. Gene profiling data of the current study revealed (i) a significant increase of IL13 mRNA levels in NP tissues, (ii) significant associations with the clinical parameters such as atopy and asthma, and (iii) a strong positive correlation between expression levels of IL13 and IL5. In line, elevated protein levels of IL13 were shown previously in a subgroup of CRS patients with polyposis [70], and IL13 is spontaneously released from NP tissue [71]. Furthermore, IL13 is considered to be a critical regulator of inflammatory and allergic responses [72] and, at the same time, as shown in mouse models, the IL13-triggered signaling cascades and downstream gene expression patterns are only partially overlapping with those induced by other Th2 cytokines [73]. Thus, our results suggest that IL13 is a candidate target for treatment of nasal polyposis.

Another potential candidate target gene strongly associated with AID and IgE molecules is CD23 (**Figure 5**). CD23, the low-affinity IgE receptor, is expressed on a variety of hematopoietic cell types [74]–[76]. CD23 has been shown to play a role in modulating the production of IgE by B cells [74], [77]. CD23 mediates numerous IgE-related immune responses (including antigen focusing) by enhancing IgE antigen complex presentation, regulating IgE synthesis, influencing cell differentiation and growth of both B and T cells, and stimulating the production of pro-inflammatory mediators from monocytes/macrophages, eosinophils, and even some types of stromal cells [76]. There are two splice forms of CD23 that can be expressed on the cell membrane; CD23a and CD23b differ at the N-terminus and display different patterns of expression and sensitivities to exogenous stimuli [78]. Thus, expression of CD23a is largely restricted to B cells, while CD23b synthesis is inducible in a variety of cells upon exogenous stimulation. The tools developed in the current study allowed us to perform comparative profiling of individual transcripts as well as the total CD23 molecule content within the same tissue. Given the significant increase of CD23 mRNA levels including the individual variants in polyp tissues, significant associations with patient's clinical parameters such as atopy and asthma, and the positive correlations between IL5–CD23/CD23b, IL13–CD23, CD23a–PAX5, and AID–CD23/CD23a it might therefore be of interest to further investigate whether CD23 might serve as drug target for the local treatment of polyposis. Currently, Lumiliximab – a chimeric macaque and human anti-CD23 monoclonal antibody – is considered as immunomodulator and is in clinical trials for the treatment of patients with chronic lymphocytic leukemia [79]. Besides leukemia, an anti-CD23 strategy has previously been considered for the treatment of asthma, allergic inflammation, and atopic dermatitis [80].

A final observation is based on the arrangement of datasets for each specimen across all genes. This approach provides the patient-specific gene expression “fingerprint”. Of particular interest are the patterns of specimens from the CRS group, which cluster within the mixed population. These specimens are distinguished based on the elevated levels of subset of genes characteristic for the NP group (e.g. IL5, CD23, IL13). The data suggest the described approach to be supportive for the patient-orientated therapeutic targeting and for the definition of new predictive factors in clinical monitoring.

Taken together, we have developed a multigene signature covering one particular disease-associated module using AID as the key gene. We further explored associations between AID and other molecules involved in the etiology of human inflammation-driven disease such as nasal polyposis: in addition to the previously highlighted biomarkers/targets [35], [70], novel players were suggested including among others IL13 and CD23 as well as genes of B cell identity. Thus, the algorithm presented herein based on the multigene signature approach, analysis of co-regularities and creation of AID-associated functional network gives an integrated view of biological processes and might be further applied to clarify role of altered AID expression in etiology of other diseases including autoimmune and malignant disorders.

## Supporting Information

Figure S1 **Expression profiles of AID in mouse and human tissues assayed by real-time PCR.** Expression levels of AID genes were normalized to the average of HKGs and shown relative (A) to salivary gland for mouse tissues and (B) to adrenal gland for human tissues. Results are shown as mean values from triplicates in one experiment ± SD and are representative of two independent experiments.
(EPS)

Figure S2 **AID expression in Raji cells assessed by immunostaining.** Cytospin preparations from Raji cells. AID expression was detected using either immunofluorescence or immunohistochemistry protocols (AID, *a*, *b*; isotype control, *c*, *d*). (a) 89.5% of cells (2532 analysed cells) were calculated to be AID-positive (TissueQuest software; TissueGnostics, Vienna, Austria); (b) 80.9% of cells (2647 analysed cells) were calculated to be AID-positive (HistoQuest software; TissueGnostics, Vienna, Austria).
(EPS)

Figure S3 **AID expression in nasal polyp tissues assessed by** ***in situ*** **immunostaining.** AID-positive cells were not detected within B cell population around the glandular structures. Scale bar: 50 µm.
(EPS)

Figure S4 **Single stained IgE-positive cells within NP.** Representative examples of single stained IgE-positive/CD138-negative cells are indicated by asterisks. The individual channels for DAPI, IgE and CD138 are shown as black/white and the merged images are in color.
(EPS)

Table S1 **Profile of study patients.** Clinical parameters including history of atopy, asthma, aspirin hypersensitivity (ASA), total IgE antibody levels, IgE specific to Staphylococcus aureus enterotoxins (SAE-IgE), levels of eosinophil cationic protein (ECP) and IL5 are indicated. BDL, below detection limit; ND, not determined.
(DOC)

Table S2 **BLAST analysis of IgG forward and reverse primers.** Gene symbol, NCBI GeneID, results of BLAST search are provided. Forward primer sequence (indicated in the Table as “Query”) was subjected to NCBI BLAST analysis. Results showed 100% homology to the IGHJ1/4/5 transcripts (indicated in the table as “Sbjct”. Tm of forward primer is 64°C and therefore this is allows to bind to the template with mismatch. IGH is an abbreviation for immunoglobulin heavy join, thereby confirmed the ability to detect IGH classes. Reverse primer sequence (indicated in the Table as “Query”) was subjected to NCBI BLAST analysis. Results showed 100% homology to the IGHG1/2/3/4 transcripts (indicated in the table as “Sbjct”; IGHG is an abbreviation for immunoglobulin heavy constant gamma), thereby confirmed the ability to detect all IgG isotypes.
(DOC)

Table S3 **Real-time PCR primers.** Gene symbol and synonyms, NCBI accession number, sequences of forward (F) and reverse (R) primers.
(DOC)

Table S4 **A panel of human genes.** Gene symbol and synonyms, NCBI accession number, short functional gene description from Gene/NCBI are provided.
(DOC)

Table S5 **Alignment of expression profiling dataset with the clinical parameters.** Comparisons between gene expression datasets (log2-transformed values) and patient's clinical parameters such as sex, history of atopy, asthma, and aspirin hypersensitivity (ASA) (from Table S1) are summarized. Analysis was done across both diseased groups. *p* value color code: *red*, statistically significant; *blue*, tendency; **bold**, passed the Holm-Bonferroni correction method for multiple comparisons.
(DOC)

Table S6 **Correlation analysis (SYSTAT program).** Pearson correlation matrix and the corresponding matrix of probabilities as well as matrix of Bonferroni probabilities are shown. The analysis was performed across all samples of two disease groups. Lines for protein transcripts are highlighted in *grey*. Color code for statistically significant co-regularities: *red* for correlation coefficient≥0.6, *p*<0.0001; *blue* for correlation coefficient<0.6, *p*<0.05.
(PDF)

Text S1 **Results.**
(DOC)
