# Correction: Activation-Induced Cytidine Deaminase (AID)-Associated Multigene Signature to Assess Impact of AID in Etiology of Diseases with Inflammatory Component

**DOI:** 10.1371/annotation/2eba0189-04c1-4dce-a85a-c7f2c7089938

**Published:** 2012-05-11

**Authors:** Diana Mechtcheriakova, Yury Sobanov, Gabriele Holtappels, Erika Bajna, Martin Svoboda, Markus Jaritz, Claus Bachert, Erika Jensen-Jarolim

There is an error in the listed affiliations for Erika Jensen-Jarolim. The correct affiliations are

1 Department of Pathophysiology and Allergy Research, Center of Pathophysiology, Infectiology and Immunology, Medical University of Vienna, Vienna, Austria,

4 Messerli Research Institute of the Medical University of Vienna, Veterinary University of Vienna and University of Vienna, Austria. 

