# Supplementary material for: Activation-Induced Cytidine Deaminase (AID)-Associated Multigene Signature to Assess Impact of AID in Etiology of Diseases with Inflammatory Component
Source: PLoS One. 2011 Oct 3;6(10):e25611. doi: 10.1371/journal.pone.0025611 (PMC3184987; doi:10.1371/journal.pone.0025611)
Supplement: Table S1 — Profile of study patients. Clinical parameters including history of atopy, asthma, aspirin hypersensitivity (ASA), total IgE antibody levels, IgE specific to Staphylococcus aureus enterotoxins (SAE-IgE), levels of eosinophil cationic protein (ECP) and IL5 are indicated. BDL, below detection limit; ND, not determined. (DOC) [file pone.0025611.s005.doc]

| **Specimens** | **SEX** | **atopy** | **asthma** | **ASA** | **IgE (kU/l)** | **SAE-IgE (kUA/l)** | **ECP (µg/l)** | **IL5 (pg/ml)** |
| --- | --- | --- | --- | --- | --- | --- | --- | --- |
| CRS 1 | M | no | no | no | 21.0 | BDL | 417.5 | 10.0 |
| CRS 2 | F | no | no | no | 13.1 | BDL | 281.7 | 10.0 |
| CRS 3 | M | no | no | no | 36.0 | BDL | 492.3 | 9.7 |
| CRS 4 | M | no | no | no | 9.5 | BDL | 1648.2 | 9.8 |
| CRS 5 | F | no | no | no | 1.9 | BDL | 321.1 | 10.0 |
| CRS 6 | M | no | no | no | 6.2 | BDL | 3148.3 | 9.9 |
| CRS 7 | M | no | no | no | 14.9 | BDL | 489.5 | BDL |
| CRS 8 | F | no | no | no | 93.0 | BDL | 6380.0 | 21.5 |
| CRS 9 | M | no | no | ND | 24.8 | BDL | 311.3 | BDL |
| CRS 10 | M | no | no | no | 210.1 | BDL | 3338.5 | BDL |
| CRS 11 | M | no | no | ND | 28.1 | BDL | 191.4 | BDL |
| CRS 12 | M | no | no | no | 342.7 | 3.9 | 16390.0 | 104.3 |
| CRS 13 | M | no | no | no | 19.8 | BDL | 781.0 | BDL |
| CRS 14 | F | no | no | no | 33.6 | BDL | 841.5 | BDL |
| CRS 15 | M | no | no | no | 13.2 | BDL | 374.0 | BDL |
| NP 1 | F | yes | no | no | 1274.8 | 7.2 | 12833.0 | 1703.9 |
| NP 2 | F | no | yes | no | 2979.4 | 17.0 | 5878.4 | 1212.4 |
| NP 3 | F | no | no | ND | 2504.2 | 27.7 | 11178.4 | 809.7 |
| NP 4 | M | yes | no | no | 623.2 | 4.2 | 12192.2 | 652.2 |
| NP 5 | M | no | yes | no | 2003.4 | 6.9 | 5997.2 | 89.7 |
| NP 6 | M | yes | yes | ND | 1137.9 | 4.3 | 5059.8 | 290.1 |
| NP 7 | M | no | yes | yes | 2581.7 | 7.7 | 11442.0 | 578.4 |
| NP 8 | M | yes | no | no | 531.7 | 5.5 | 7267.2 | 357.1 |
| NP 9 | M | yes | yes | no | 957.0 | 24.9 | 20130.0 | 418.6 |
| NP 10 | F | no | no | no | 126.5 | BDL | 7590.0 | 31.0 |
| NP 11 | M | yes | yes | yes | 451.6 | 5.3 | 20680.0 | 285.6 |
| NP 12 | F | yes | no | no | 1391.5 | BDL | 17710.0 | 1017.0 |
| NP 13 | F | no | no | no | 398.8 | BDL | 5775.0 | 117.5 |
| NP 14 | M | yes | no | no | 53.4 | BDL | 819.5 | BDL |
| NP 15 | M | no | yes | ND | 1463.0 | 8.6 | 20460.0 | 169.4 |
| NP 16 | F | no | yes | no | 280.5 | 6.6 | 11550.0 | 59.0 |
| NP 17 | M | yes | yes | ND | 131.5 | BDL | 25960.0 | 110.9 |
| NP 18 | M | yes | no | no | 4031.5 | 7.0 | 682.0 | 485.8 |
