# Supplementary material for: Activation-Induced Cytidine Deaminase (AID)-Associated Multigene Signature to Assess Impact of AID in Etiology of Diseases with Inflammatory Component
Source: PLoS One. 2011 Oct 3;6(10):e25611. doi: 10.1371/journal.pone.0025611 (PMC3184987; doi:10.1371/journal.pone.0025611)
Supplement: Table S2 — BLAST analysis of IgG forward and reverse primers. Gene symbol, NCBI GeneID, results of BLAST search are provided. Forward primer sequence (indicated in the Table as “Query”) was subjected to NCBI BLAST analysis. Results showed 100% homology to the IGHJ1/4/5 transcripts (indicated in the table as “Sbjct”. Tm of forward primer is 64°C and therefore this is allows to bind to the template with mismatch. IGH is an abbreviation for immunoglobulin heavy join, thereby confirmed the ability to detect IGH classes. Reverse primer sequence (indicated in the Table as “Query”) was subjected to NCBI BLAST analysis. Results showed 100% homology to the IGHG1/2/3/4 transcripts (indicated in the table as “Sbjct”; IGHG is an abbreviation for immunoglobulin heavy constant gamma), thereby confirmed the ability to detect all IgG isotypes. (DOC) [file pone.0025611.s006.doc]

| gene | GeneID | Forward Primer Sequence |
| --- | --- | --- |
| IGHJ1 | [28483](http://www.ncbi.nlm.nih.gov/entrez/query.fcgi?db=gene&cmd=Retrieve&dopt=full_report&list_uids=28483) | Query 1 ACCCTGGTCACCGTCTCCTCAG 22  ||||||||||||||||||||||  Sbjct 87332683 ACCCTGGTCACCGTCTCCTCAG 87332662 |
| IGHJ4 | [28477](http://www.ncbi.nlm.nih.gov/entrez/query.fcgi?db=gene&cmd=Retrieve&dopt=full_report&list_uids=28477) | Query 1 ACCCTGGTCACCGTCTCCTCAG 22  ||||||||||||||||||||||  Sbjct 87331491 ACCCTGGTCACCGTCTCCTCAG 87331470 |
| IGHJ5 | [28476](http://www.ncbi.nlm.nih.gov/entrez/query.fcgi?db=gene&cmd=Retrieve&dopt=full_report&list_uids=28476) | Query 1 ACCCTGGTCACCGTCTCCTCAG 22  ||||||||||||||||||||||  Sbjct 87331090 ACCCTGGTCACCGTCTCCTCAG 87331069 |
| IGHJ2 | [28481](http://www.ncbi.nlm.nih.gov/entrez/query.fcgi?db=gene&cmd=Retrieve&dopt=full_report&list_uids=28481) | Query 1 ACCCTGGTCACCGTCTCCTCAG 22  ||||||||||| ||||||||||  Sbjct 87332475 ACCCTGGTCACTGTCTCCTCAG 87332454 |
| IGHJ6 | [28475](http://www.ncbi.nlm.nih.gov/entrez/query.fcgi?db=gene&cmd=Retrieve&dopt=full_report&list_uids=28475) | Query 1 ACCCTGGTCACCGTCTCCTCAG 22  ||| |||||||||||||||||  Sbjct 87330472 ACCACGGTCACCGTCTCCTCAG 87330453 |
| IGHJ3 | [28479](http://www.ncbi.nlm.nih.gov/entrez/query.fcgi?db=gene&cmd=Retrieve&dopt=full_report&list_uids=28479) | Query 1 ACCCTGGTCACCGTCTCCTCAG 22  || ||||||||||||| ||||  Sbjct 87331863 ACAATGGTCACCGTCTCTTCAG 87331842 |

| gene | GeneID | Reverse Primer Sequence |
| --- | --- | --- |
| IGHG1 | [3500](http://www.ncbi.nlm.nih.gov/entrez/query.fcgi?db=gene&cmd=Retrieve&dopt=full_report&list_uids=3500) | Query 1 GTTCCACGACACCGTCACC 19  |||||||||||||||||||  Sbjct 87210328 GTTCCACGACACCGTCACC 87210346 |
| IGHG2 | [3501](http://www.ncbi.nlm.nih.gov/entrez/query.fcgi?db=gene&cmd=Retrieve&dopt=full_report&list_uids=3501) | Query 1 GTTCCACGACACCGTCACC 19  |||||||||||||||||||  Sbjct 87112047 GTTCCACGACACCGTCACC 87112065 |
| IGHG3 | [3502](http://www.ncbi.nlm.nih.gov/entrez/query.fcgi?db=gene&cmd=Retrieve&dopt=full_report&list_uids=3502) | Query 1 GTTCCACGACACCGTCACC 19  |||||||||||||||||||  Sbjct 87238663 GTTCCACGACACCGTCACC 87238681 |
| IGHG4 | [3503](http://www.ncbi.nlm.nih.gov/entrez/query.fcgi?db=gene&cmd=Retrieve&dopt=full_report&list_uids=3503) | Query 1 GTTCCACGACACCGTCACC 19  |||||||||||||||||||  Sbjct 87093323 GTTCCACGACACCGTCACC 87093341 |
