# Supplementary material for: Activation-Induced Cytidine Deaminase (AID)-Associated Multigene Signature to Assess Impact of AID in Etiology of Diseases with Inflammatory Component
Source: PLoS One. 2011 Oct 3;6(10):e25611. doi: 10.1371/journal.pone.0025611 (PMC3184987; doi:10.1371/journal.pone.0025611)
Supplement: Table S3 — Real-time PCR primers. Gene symbol and synonyms, NCBI accession number, sequences of forward (F) and reverse (R) primers. (DOC) [file pone.0025611.s007.doc]

| **No** | **Symbol** | | **Synonym** | **Accession number** | **sequences of primers** |
| --- | --- | --- | --- | --- | --- |
| 1 | AID | AICDA  AIDex4 | | [NM_020661](http://www.ncbi.nlm.nih.gov/entrez/viewer.fcgi?val=NM_020661.1) | F:GGACTTTGGTTATCTTCGCAATAAG  R:GTCGGGCACAGTCGTAGCA  F:ATAGCCATCATGACCTTCAAAGATT  R:GCCGAAGCTGTCTGGAGAGA |
| 2 | AIDdel | AIDdelEx4 | | [AY536517](http://www.ncbi.nlm.nih.gov/entrez/viewer.fcgi?db=nucleotide&val=46403718) | F:ATAGCCATCATGACCTTCAAAGATT  R:GCCGAAGCTGTCTGGAGAGA |
| 3 | IL5 | TRF | | [NM_000879](http://www.ncbi.nlm.nih.gov/entrez/query.fcgi?cmd=Retrieve&db=Nucleotide&list_uids=28559032&dopt=GenBank) | F:TGCTGATAGCCAATGAGACTCTGA  R:AGTGTGCCTATTCCCTGAAAGATT |
| 4 | IL13 | ALRH  BHR1 | | [NM_002188](http://www.ncbi.nlm.nih.gov/entrez/viewer.fcgi?val=NM_002188.2) | F:ACCTGACAGCTGGCATGTACTG  R:TGGGTCTTCTCGATGGCACT |
| 5 | CD3 | CD3E | | [NM_000733](http://www.ncbi.nlm.nih.gov/entrez/viewer.fcgi?db=nuccore&val=166362733) | F:CTGGCGGCAGGCAAAGG  R:TTCCGGATGGGCTCATAGTCT |
| 6 | CD14 |  | | [NM_001040021](http://www.ncbi.nlm.nih.gov/nuccore/NM_001040021.2) | F:CGGAAGACTTATCGACCATGGAG  R:TCGCAGAGACGTGCACCAG |
| 7 | CD19 | B4 | | [NM_001770](http://www.ncbi.nlm.nih.gov/nuccore/NM_001770.5) | F:GGCAACCTGACCATGTCATTC  R:TCAGCAGCCAGTGCCATAGT |
| 8 | CD23 | FCER2 | | [NM_002002](http://www.ncbi.nlm.nih.gov/entrez/viewer.fcgi?db=nucleotide&val=34147598) | F:AGGTGTCCAGCGGCTTTGT  R:AGCACTTCCGTTGGAAATTGA |
| 9 | CD23a | FCER2 | | [NM_002002](http://www.ncbi.nlm.nih.gov/entrez/viewer.fcgi?db=nucleotide&val=34147598) | F:CACAGGCTCCAAACTCCACTAAG  R:CCCGATGATGGAGCACTCA |
| 10 | CD23b | FCER2 | |  | F:CCAAGCCAGGAGATCGAGG  R:GCAGCACGATCTGAGTCCCA |
| 11 | CD86 | B70  B7-2 | | [NM_175862](http://www.ncbi.nlm.nih.gov/nuccore/NM_175862.3) | F:ACAAAAAGCCCACAGGAATGAT  R:TCAGGTTGACTGAAGTTAGCAAGC |
| 12 | IgM | IGHM | |  | F:ACCCTGGTCACCGTCTCCTCAG  R:GAAGTCCTGTGCGAGGCAG |
| 13 | IgG | IGHG1 | |  | F:ACCCTGGTCACCGTCTCCTCAG  R:GTTCCACGACACCGTCACC |
| 14 | IgE | IGHE | |  | F:ACCCTGGTCACCGTCTCCTCAG  R:CAGAGTCACGGAGGTGGCATT |
| 15 | PAX5 | BSAP | | [NM_016734](http://www.ncbi.nlm.nih.gov/entrez/viewer.fcgi?val=NM_016734.1) | F:CGTACAACGACTCCTGGAGGTTC  R:GGCGGCAGCGCTATAATAGTA |
| 16 | IRF8 | ICSBP1 | | [NM_002163](http://www.ncbi.nlm.nih.gov/entrez/viewer.fcgi?val=NM_002163.2) | F:CTGGACATTTCCGAGCCATAC  R:TGCCACGCCTAGTTTGCAT |
| 17 | ID2 |  | | [NM_002166](http://www.ncbi.nlm.nih.gov/entrez/viewer.fcgi?val=NM_002166.4) | F:TGTGGCTGAATAAGCGGTGTT  R:TCAGCACTTAAAAGATTCCGTGAA |
| 18 | ID3 |  | | [NM_002167](http://www.ncbi.nlm.nih.gov/entrez/viewer.fcgi?val=NM_002167.3) | F:GCTCACTCCGGAACTTGTCATC  R:CCAGCACCTGCGTTCTGGA |
| 19 | FcεRIa | FcERI | | [NM_002001](http://www.ncbi.nlm.nih.gov/entrez/viewer.fcgi?val=NM_002001.2) | F:CATGGAATCCCCTACTCTACTGTGT  R:CCTTAGGTTTCTGAGGGACTGCTA |
| 20 | FcεRIb | MS4A2 | | [NM_000139](http://www.ncbi.nlm.nih.gov/entrez/viewer.fcgi?val=NM_000139.2) | F:GAGAAATGCAACATATCTGGTGAGAG  R:AGGTTGATGATCAGGATGGTAATTC |
| 21 | FcεRIg |  | | [NM_004106](http://www.ncbi.nlm.nih.gov/entrez/viewer.fcgi?val=NM_004106.1) | F:TCTACTGTCGACTGAAGGTAATCCA  R:GAGTCTCGTAAGTCTCCTGGTTCCT |
| 22 | EGR-1 | KROX24  NGFIA  ZIF268 | | [NM_001964](http://www.ncbi.nlm.nih.gov/entrez/viewer.fcgi?val=NM_001964.2) | F:TGAACGCAAGAGGCATACCA  R:AAGCAGGGGGAACAGAGGA |
| 23 | EGR-2 | KROX20 | | [NM_000399](http://www.ncbi.nlm.nih.gov/nuccore/NM_000399.3) | F:GCAAATGATGACCGCCAAG  R:GGATATGGGAGATCCAACGAC |
| 24 | EGR-3 | PILOT | | [NM_004430](http://www.ncbi.nlm.nih.gov/nuccore/NM_004430.2) | F:CAATCTGTACCCCGAGGAG  R:TGGTCAGACCGATGTCCATTAC |
| 25 | CD21L | CR2, transcript variant 1 | | [NM_001006658](http://www.ncbi.nlm.nih.gov/entrez/viewer.fcgi?db=nucleotide&id=260099695) | F:CCCACGGTGTGAAGAAACAT  R:AACTAGCTCCACACGTGAACCA |
| 26 | EF1A | EEF1A1 | | [NM_001402](http://www.ncbi.nlm.nih.gov/nuccore/NM_001402.5) | F:ATTACAGGGACATCTCAGGCTGAC  R:CATTCTTGGAGATACCAGCTTCAA |
| 27 | b2M |  | | [NM_004048](http://www.ncbi.nlm.nih.gov/nuccore/NM_004048.2) | F:GATGAGTATGCCTGCCGTGTG  R:CAATCCAAATGCGGCATCT |
| 28 | UBC |  | | [NM_021009](http://www.ncbi.nlm.nih.gov/nuccore/NM_021009.4) | F:ATTTGGGTCGCAGTTCTTG  R:TGCCTTGACATTCTCGATGGT |
| 29 | ACTB |  | | [NM_001101](http://www.ncbi.nlm.nih.gov/nuccore/NM_001101.3) | F:TGGCTCCCGAGGAGCAC  R:TTGAAGGTCTCAAACATGATCTGG |
