# Supplementary material for: Activation-Induced Cytidine Deaminase (AID)-Associated Multigene Signature to Assess Impact of AID in Etiology of Diseases with Inflammatory Component
Source: PLoS One. 2011 Oct 3;6(10):e25611. doi: 10.1371/journal.pone.0025611 (PMC3184987; doi:10.1371/journal.pone.0025611)
Supplement: Table S4 — A panel of human genes. Gene symbol and synonyms, NCBI accession number, short functional gene description from Gene/NCBI are provided. (DOC) [file pone.0025611.s008.doc]

| **Symbol** | **Synonym** | **Accession number** | **Short functional description** |
| --- | --- | --- | --- |
| AID | AICDA | [NM_020661](http://www.ncbi.nlm.nih.gov/entrez/viewer.fcgi?val=NM_020661.1) | Activation-induced cytidine deaminase functions by deaminating dC residues in DNA. |
| AID-Δex4 | AID delEx4 | [AY536517](http://www.ncbi.nlm.nih.gov/entrez/viewer.fcgi?db=nucleotide&val=46403718) | The alternative AID splice variant; displays different activities ranging: inactivation of CSR and heightened SHM activity. |
| IL5 | TRF | [NM_000879](http://www.ncbi.nlm.nih.gov/entrez/query.fcgi?cmd=Retrieve&db=Nucleotide&list_uids=28559032&dopt=GenBank) | T-cell replacing factor; B cell differentiation factor I; eosinophil differentiation factor. |
| IL13 | ALRH BHR1 | [NM_002188](http://www.ncbi.nlm.nih.gov/entrez/viewer.fcgi?val=NM_002188.2) | This gene encodes an immunoregulatory cytokine produced primarily by activated Th2 cells. This cytokine is involved in several stages of B-cell maturation and differentiation; promotes IgE isotype switching of B cells. |
| CD3 | CD3E | [NM_000733](http://www.ncbi.nlm.nih.gov/entrez/viewer.fcgi?db=nuccore&val=166362733) | CD3-epsilon polypeptide together with CD3-gamma, -delta and -zeta, and the T-cell receptor heterodimers, forms the T-cell receptor-CD3 complex. |
| CD14 |  | [NM_001040021](http://www.ncbi.nlm.nih.gov/nuccore/NM_001040021.2) | This protein is a surface antigen that is preferentially expressed on monocytes/macrophages. It cooperates with other proteins to mediate the innate immune response to bacterial lipopolysaccharide. |
| CD19 | B4 | [NM_001770](http://www.ncbi.nlm.nih.gov/nuccore/NM_001770.5) | Surface molecule which assembles with the antigen receptor of B lymphocytes in order to decrease the threshold for antigen receptor-dependent stimulation. |
| CD23 | FCER2 | [NM_002002](http://www.ncbi.nlm.nih.gov/entrez/viewer.fcgi?db=nucleotide&val=34147598) | This C-type lectin is the low-affinity receptor for IgE. It is a key molecule for B-cell activation and growth. |
| CD86 | B70  B7-2 | [NM_175862](http://www.ncbi.nlm.nih.gov/nuccore/NM_175862.3) | This protein is expressed by antigen-presenting cells, and it is the ligand for two proteins at the cell surface of T cells, CD28 antigen and CTLA-4. Binding of this protein with CD28 antigen is a costimulatory signal for activation of the T-cell. Binding of this protein with CTLA-4 negatively regulates T-cell activation and diminishes the immune response. |
| IgM | IGHM | [X17115](http://www.ncbi.nlm.nih.gov/nuccore/33450) | Immunoglobulin (Ig) mu constant heavy chain and variable (V) domain. |
| IgG | IgG1  IgG2  IgG3  IgG4 | [J00228](http://www.ncbi.nlm.nih.gov/nuccore/184739) | Immunoglobulin (Ig) gamma constant heavy chain and variable domain. |
| IgE | IGHE | [X95746.1](http://www.ncbi.nlm.nih.gov/sites/entrez?cmd=Retrieve&db=Nucleotide&list_uids=1514574&dopt=GenBank&RID=1MJDKR4U01S&log$=nucltop&blast_rank=17) | Immunoglobulin (Ig) epsilon chain constant heavy chain and variable domain. |
| Pax5 | BSAP | [NM_016734](http://www.ncbi.nlm.nih.gov/entrez/viewer.fcgi?val=NM_016734.1) | B-cell lineage specific activator protein/transcription factor that is expressed at early, but not late stages of B-cell differentiation. Deregulation of transcription of this gene contributes to the pathogenesis of lymphomas. |
| IRF8 | ICSBP1 | [NM_002163](http://www.ncbi.nlm.nih.gov/entrez/viewer.fcgi?val=NM_002163.2) | Interferon consensus sequence-binding protein (ICSBP) is a transcription factor; involves in regulation of Spi-1/PU.1 and STAT1 transcriptional activities. |
| ID2 |  | [NM_002166](http://www.ncbi.nlm.nih.gov/entrez/viewer.fcgi?val=NM_002166.4) | Belongs to the inhibitor of DNA binding (ID) family. Id2 KO mice lack lymph nodes and Peyer patches and have reduced numbers of NK cells. These mice secreted increasing amounts of IgE in sera. |
| ID3 |  | [NM_002167](http://www.ncbi.nlm.nih.gov/entrez/viewer.fcgi?val=NM_002167.3) | ID3 is an inhibitor of E proteins, such as E2A. Proposed to be involved in autoimmune development. Id3 -/- mice revealed lymphocytic infiltration in the lachrymal and salivary glands in the absence of infection; CD4, CD8 T cells, and B cells in the infiltrates expressed both IFNγ and IL-4. |
| FcεRIa | FcERI | [NM_002001](http://www.ncbi.nlm.nih.gov/entrez/viewer.fcgi?val=NM_002001.2) | Fc fragment of IgE receptor, alpha polypeptide. The IgE receptor plays a central role in allergic disease, coupling allergen and mast cell to initiate the inflammatory and immediate hypersensitivity responses. |
| FcεRIb | MS4A2 | [NM_000139](http://www.ncbi.nlm.nih.gov/entrez/viewer.fcgi?val=NM_000139.2) | This gene encodes the beta subunit of the high affinity IgE receptor. |
| FcεRIg |  | [NM_004106](http://www.ncbi.nlm.nih.gov/entrez/viewer.fcgi?val=NM_004106.1) | This gene encodes the gamma subunit of the high affinity IgE receptor. |
| EGR-1 | KROX24  NGFIA  ZIF268 | [NM_001964](http://www.ncbi.nlm.nih.gov/entrez/viewer.fcgi?val=NM_001964.2) | EGR-1 is an early growth response gene. It is a nuclear protein and functions as a transcriptional regulator. Egr1 -/- mice showed an increase serum level of IgE. |
| EGR-2 | KROX20 | [NM_000399](http://www.ncbi.nlm.nih.gov/nuccore/NM_000399.3) | EGR-2 is able significantly suppress the growth of cancer cells. Egr-2 and Egr-3 are associated with induction of anergy in mouse T cells. Overexpression of Egr-2 or Egr-3 inhibited Il2 production |
| EGR-3 | PILOT | [NM_004430](http://www.ncbi.nlm.nih.gov/nuccore/NM_004430.2) | EGR-3 like EGR-1 and EGR-2 is an immediate-early growth response gene, which is induced by mitogenic stimulation. Egr3 -/- mice were resistant to peptide-induced immunologic tolerance. EGR3 like EGR2 are involved in promoting a T-cell receptor-induced negative regulatory genetic program. |
| CD21L | CR2, transcript variant 1 | [NM_001006658](http://www.ncbi.nlm.nih.gov/entrez/viewer.fcgi?db=nucleotide&id=260099695) | Complement component receptor-2 (CR2) is the membrane protein on B and T lymphocytes to which the Epstein-Barr virus (EBV) binds. CD21L (long) isoform is expressed specifically by follicular dendritic cells. |
