# Supplementary material for: Activation-Induced Cytidine Deaminase (AID)-Associated Multigene Signature to Assess Impact of AID in Etiology of Diseases with Inflammatory Component
Source: PLoS One. 2011 Oct 3;6(10):e25611. doi: 10.1371/journal.pone.0025611 (PMC3184987; doi:10.1371/journal.pone.0025611)
Supplement: Table S5 — Alignment of expression profiling dataset with the clinical parameters. Comparisons between gene expression datasets (log2-transformed values) and patient's clinical parameters such as sex, history of atopy, asthma, and aspirin hypersensitivity (ASA) (from Table S1) are summarized. Analysis was done across both diseased groups. p value color code: red, statistically significant; blue, tendency; bold, passed the Holm-Bonferroni correction method for multiple comparisons. (DOC) [file pone.0025611.s009.doc]

|  | |  |  |  |
| --- | --- | --- | --- | --- |
|  | Sex | Atopy | Asthma | ASA |
| AID | .393 | .134 | .051 | .752 |
| CD23 | .762 | **.001** | **.002** | .152 |
| CD23a | .557 | .064 | **.003** | .827 |
| CD23b | .766 | .004 | **.001** | .138 |
| IL13 | .744 | .020 | **.003** | .340 |
| IL5 | .327 | .015 | .004 | .205 |
| CD19 | .565 | **.003** | .022 | .503 |
| CD86 | .840 | .006 | .085 | .078 |
| CD14 | .225 | .025 | .021 | .459 |
| IgG | .605 | .019 | **.002** | .114 |
| IgE | .836 | .006 | .052 | .108 |
| FceRIa | .932 | .005 | .264 | .130 |
| FceRIb | .544 | .126 | .165 | .790 |
| FceRIg | .615 | .007 | .010 | .160 |
| IRF8 | .147 | .134 | .157 | .546 |
| ID3 | .836 | .011 | .078 | .363 |
