# Supplementary material for: Activation-Induced Cytidine Deaminase (AID)-Associated Multigene Signature to Assess Impact of AID in Etiology of Diseases with Inflammatory Component
Source: PLoS One. 2011 Oct 3;6(10):e25611. doi: 10.1371/journal.pone.0025611 (PMC3184987; doi:10.1371/journal.pone.0025611)
Supplement: Table S6 — Correlation analysis (SYSTAT program). Pearson correlation matrix and the corresponding matrix of probabilities as well as matrix of Bonferroni probabilities are shown. The analysis was performed across all samples of two disease groups. Lines for protein transcripts are highlighted in grey. Color code for statistically significant co-regularities: red for correlation coefficient≥0.6, p<0.0001; blue for correlation coefficient<0.6, p<0.05. (PDF) [file pone.0025611.s010.pdf]

Pearson Correlation Matrix (SYSTAT 12 program; analysis across all samples combining both diseased groups)

	AID	AIDDELTA	IL13	IL5	CD14	CD19	CD3E	CD86	IgM	IgG	IgE	CD23	CD23a	CD23b	FCERIA	FCERIB	FCERIG	IRF8	ID2	ID3	PAX5	EGR_1	EGR_2	EGR_3	CD21L	ECP	IL5_P	SAE_IGE	IGE_P
AID	1																												
AIDDELTA	0.151	1																											
IL13	0.649	0.09	1																										
IL5	0.692	0.028	0.865	1																									
CD14	0.548	0.468	0.299	0.351	1																								
CD19	0.656	0.16	0.513	0.542	0.497	1																							
CD3E	0.418	0.01	0.183	0.309	0.404	0.506	1																						
CD86	0.571	0.387	0.505	0.492	0.79	0.522	0.411	1																					
IgM	-0.261	-0.115	-0.129	-0.096	-0.019	-0.144	0.285	0.017	1																				
IgG	0.471	0.015	0.497	0.451	0.459	0.795	0.38	0.418	-0.198	1																			
IgE	0.417	0.031	0.726	0.501	0.383	0.454	0.303	0.478	-0.054	0.615	1																		
CD23	0.683	0.227	0.838	0.737	0.55	0.688	0.363	0.733	-0.128	0.654	0.667	1																	
CD23a	0.709	0.016	0.595	0.516	0.464	0.518	0.218	0.657	-0.174	0.408	0.413	0.727	1																
CD23b	0.551	0.324	0.774	0.638	0.485	0.675	0.29	0.595	-0.121	0.717	0.621	0.935	0.526	1															
FCERIA	0.273	0.684	0.345	0.304	0.524	0.238	0.149	0.651	0.02	0.158	0.264	0.49	0.193	0.507	1														
FCERIB	0.421	0.031	0.688	0.571	0.176	0.4	-0.011	0.431	-0.085	0.404	0.352	0.666	0.545	0.683	0.395	1													
FCERIG	0.494	0.553	0.484	0.411	0.804	0.543	0.42	0.879	-0.07	0.506	0.449	0.727	0.471	0.686	0.672	0.358	1												
IRF8	0.215	0.078	0.116	0.07	0.468	0.351	0.479	0.572	0.39	0.236	0.252	0.246	0.389	0.18	0.292	0.135	0.434	1											
ID2	-0.142	0.285	-0.38	-0.32	-0.026	-0.216	0.084	-0.074	0.02	-0.365	-0.292	-0.348	-0.261	-0.313	0.043	-0.383	-0.074	0.035	1										
ID3	-0.303	-0.211	-0.414	-0.4	-0.147	-0.384	-0.123	-0.292	0.188	-0.298	-0.238	-0.331	-0.155	-0.359	-0.369	-0.428	-0.357	-0.109	0.335	1									
PAX5	0.601	0.106	0.389	0.362	0.545	0.548	0.534	0.614	0.099	0.388	0.352	0.494	0.779	0.332	0.219	0.311	0.453	0.664	-0.009	-0.109	1								
EGR_1	0.084	0.539	-0.244	-0.187	0.221	0.133	0.189	0.143	0.032	-0.166	-0.161	-0.096	-0.063	-0.073	0.253	-0.171	0.153	0.133	0.596	0.321	0.15	1							
EGR_2	0.038	0.268	-0.169	-0.077	0.031	-0.024	0.354	0.099	0.134	-0.122	-0.113	-0.052	-0.131	-0.006	0.264	-0.083	0.107	0.141	0.545	0.157	0.02	0.626	1						
EGR_3	-0.129	0.084	-0.319	-0.219	-0.208	-0.252	0.049	-0.14	0.074	-0.27	-0.287	-0.279	-0.231	-0.227	0.03	-0.237	-0.159	-0.016	0.419	0.192	-0.194	0.434	0.863	1					
CD21L	-0.065	-0.018	-0.169	-0.137	0.131	-0.135	-0.193	-0.047	0.107	-0.148	-0.134	-0.192	-0.127	-0.164	0.064	0.048	-0.073	0.167	-0.147	-0.132	-0.095	-0.13	-0.11	-0.052	1				
ECP	0.182	0.175	0.434	0.453	0.221	0.28	0.092	0.153	-0.197	0.312	0.255	0.394	0.074	0.4	0.261	0.323	0.396	-0.112	-0.475	-0.518	-0.057	-0.249	-0.112	-0.183	0.289	1			
IL5_P	0.321	-0.074	0.488	0.386	0.237	0.267	0.219	0.366	0.004	0.266	0.518	0.511	0.432	0.381	0.214	0.453	0.258	0.107	-0.448	-0.247	0.235	-0.193	-0.074	-0.218	0.095	0.383	1		
SAE_IgE	0.307	-0.08	0.508	0.543	0.031	0.242	0.02	0.16	-0.198	0.221	0.383	0.319	0.459	0.167	0.003	0.271	0.037	0.064	-0.421	-0.338	0.334	-0.266	-0.235	-0.224	0.055	0.412	0.531	1	
IgE_P	0.504	-0.098	0.58	0.527	0.519	0.384	0.213	0.557	-0.207	0.528	0.747	0.55	0.605	0.411	0.17	0.401	0.383	0.328	-0.384	-0.309	0.483	-0.273	-0.241	-0.352	0.041	0.204	0.626	0.63	1

Matrix of Probabilities

	AID	AIDDELTA	IL13	IL5	CD14	CD19	CD3E	CD86	IgM	IgG	IgE	CD23	CD23a	CD23b	FCERIA	FCERIB	FCERIG	IRF8	ID2	ID3	PAX5	EGR_1	EGR_2	EGR_3	CD21L	ECP	IL5_P	SAE_IGE	IGE_P
AID	0.000																												
AIDDELTA	0.394	0.000																											
IL13	0.000	0.614	0.000																										
IL5	0.000	0.877	0.000	0.000																									
CD14	0.001	0.005	0.086	0.042	0.000																								
CD19	0.000	0.367	0.002	0.001	0.003	0.000																							
CD3E	0.014	0.954	0.300	0.075	0.018	0.002	0.000																						
CD86	0.000	0.024	0.002	0.003	0.000	0.002	0.016	0.000																					
IgM	0.136	0.518	0.468	0.591	0.916	0.416	0.102	0.924	0.000																				
IgG	0.005	0.931	0.003	0.007	0.006	0.000	0.027	0.014	0.262	0.000																			
IgE	0.014	0.862	0.000	0.003	0.025	0.007	0.081	0.004	0.761	0.000	0.000																		
CD23	0.000	0.196	0.000	0.000	0.001	0.000	0.035	0.000	0.469	0.000	0.000	0.000																	
CD23a	0.000	0.928	0.000	0.002	0.006	0.002	0.217	0.000	0.326	0.017	0.015	0.000	0.000																
CD23b	0.001	0.062	0.000	0.000	0.004	0.000	0.096	0.000	0.495	0.000	0.000	0.000	0.001	0.000															
FCERIA	0.118	0.000	0.046	0.080	0.001	0.175	0.401	0.000	0.912	0.372	0.131	0.003	0.273	0.002	0.000														
FCERIB	0.013	0.860	0.000	0.000	0.319	0.019	0.949	0.011	0.634	0.018	0.041	0.000	0.001	0.000	0.021	0.000													
FCERIG	0.003	0.001	0.004	0.016	0.000	0.001	0.014	0.000	0.693	0.002	0.008	0.000	0.005	0.000	0.000	0.038	0.000												
IRF8	0.222	0.662	0.514	0.694	0.005	0.042	0.004	0.000	0.023	0.178	0.151	0.161	0.023	0.309	0.094	0.447	0.010	0.000											
ID2	0.425	0.103	0.027	0.065	0.882	0.220	0.635	0.676	0.910	0.034	0.094	0.044	0.136	0.071	0.809	0.025	0.679	0.844	0.000										
ID3	0.081	0.230	0.015	0.019	0.406	0.025	0.488	0.093	0.287	0.087	0.175	0.056	0.380	0.037	0.032	0.011	0.038	0.540	0.053	0.000									
PAX5	0.000	0.550	0.023	0.035	0.001	0.001	0.001	0.000	0.579	0.023	0.041	0.003	0.000	0.055	0.213	0.074	0.007	0.000	0.961	0.540	0.000								
EGR_1	0.635	0.001	0.165	0.291	0.209	0.452	0.284	0.421	0.857	0.349	0.363	0.591	0.723	0.680	0.149	0.334	0.388	0.454	0.000	0.064	0.396	0.000							
EGR_2	0.830	0.126	0.338	0.666	0.861	0.893	0.040	0.576	0.450	0.493	0.524	0.769	0.459	0.973	0.131	0.640	0.546	0.426	0.001	0.377	0.909	0.000	0.000						
EGR_3	0.469	0.638	0.066	0.212	0.238	0.151	0.783	0.428	0.678	0.123	0.100	0.110	0.190	0.196	0.867	0.177	0.369	0.927	0.014	0.277	0.271	0.010	0.000	0.000					
CD21L	0.714	0.920	0.339	0.439	0.459	0.445	0.275	0.792	0.547	0.405	0.451	0.276	0.474	0.354	0.718	0.788	0.681	0.344	0.406	0.456	0.595	0.465	0.534	0.769	0.000				
ECP	0.302	0.322	0.010	0.007	0.209	0.109	0.605	0.389	0.265	0.072	0.146	0.021	0.676	0.019	0.136	0.062	0.020	0.529	0.004	0.002	0.749	0.156	0.529	0.299	0.097	0.000			
IL5_P	0.064	0.676	0.003	0.024	0.177	0.126	0.212	0.033	0.981	0.129	0.002	0.002	0.011	0.026	0.225	0.007	0.141	0.547	0.008	0.160	0.181	0.273	0.676	0.216	0.593	0.025	0.000		
SAE_IgE	0.077	0.653	0.002	0.001	0.862	0.168	0.911	0.366	0.261	0.209	0.026	0.066	0.006	0.344	0.987	0.122	0.834	0.718	0.013	0.051	0.053	0.128	0.181	0.203	0.759	0.015	0.001	0.000	
IgE_P	0.002	0.583	0.000	0.001	0.002	0.025	0.226	0.001	0.241	0.001	0.000	0.001	0.000	0.016	0.337	0.019	0.025	0.058	0.025	0.075	0.004	0.118	0.170	0.041	0.819	0.247	0.000	0.000	0.000

Matrix of Bonferroni Probabilities

	AID	AIDDELTA	IL13	IL5	CD14	CD19	CD3E	CD86	IgM	IgG	IgE	CD23	CD23a	CD23b	FCERIA	FCERIB	FCERIG	IRF8	ID2	ID3	PAX5	EGR_1	EGR_2	EGR_3	CD21L	ECP	IL5_P	SAE_IGE	IGE_P
AID	0.000																												
AIDDELTA	1.000	0.000																											
IL13	0.013	1.000	0.000																										
IL5	0.002	1.000	0.000	0.000																									
CD14	0.319	1.000	1.000	1.000	0.000																								
CD19	0.010	1.000	0.783	0.381	1.000	0.000																							
CD3E	1.000	1.000	1.000	1.000	1.000	0.910	0.000																						
CD86	0.170	1.000	0.940	1.000	0.000	0.626	1.000	0.000																					
IgM	1.000	1.000	1.000	1.000	1.000	1.000	1.000	1.000	0.000																				
IgG	1.000	1.000	1.000	1.000	1.000	0.000	1.000	1.000	1.000	0.000																			
IgE	1.000	1.000	0.000	1.000	1.000	1.000	1.000	1.000	1.000	0.045	0.000																		
CD23	0.003	1.000	0.000	0.000	0.302	0.003	1.000	0.000	1.000	0.011	0.007	0.000																	
CD23a	0.001	1.000	0.083	0.717	1.000	0.694	1.000	0.010	1.000	1.000	1.000	0.000	0.000																
CD23b	0.299	1.000	0.000	0.020	1.000	0.005	1.000	0.083	1.000	0.001	0.037	0.000	0.562	0.000															
FCERIA	1.000	0.003	1.000	1.000	0.602	1.000	1.000	0.013	1.000	1.000	1.000	1.000	1.000	0.901	0.000														
FCERIB	1.000	1.000	0.003	0.173	1.000	1.000	1.000	1.000	1.000	1.000	1.000	0.007	0.345	0.003	1.000	0.000													
FCERIG	1.000	0.281	1.000	1.000	0.000	0.366	1.000	0.000	1.000	0.919	1.000	0.000	1.000	0.003	0.005	1.000	0.000												
IRF8	1.000	1.000	1.000	1.000	1.000	1.000	1.000	0.165	1.000	1.000	1.000	1.000	1.000	1.000	1.000	1.000	1.000	0.000											
ID2	1.000	1.000	1.000	1.000	1.000	1.000	1.000	1.000	1.000	1.000	1.000	1.000	1.000	1.000	1.000	1.000	1.000	1.000	0.000										
ID3	1.000	1.000	1.000	1.000	1.000	1.000	1.000	1.000	1.000	1.000	1.000	1.000	1.000	1.000	1.000	1.000	1.000	1.000	1.000	0.000									

PAX5	0.070	1.000	1.000	1.000	0.349	0.322	0.468	0.046	1.000	1.000	1.000	1.000	0.000	1.000	1.000	1.000	1.000	0.008	1.000	1.000	0.000								
EGR_1	1.000	0.408	1.000	1.000	1.000	1.000	1.000	1.000	1.000	1.000	1.000	1.000	1.000	1.000	1.000	1.000	1.000	1.000	0.080	1.000	1.000	0.000							
EGR_2	1.000	1.000	1.000	1.000	1.000	1.000	1.000	1.000	1.000	1.000	1.000	1.000	1.000	1.000	1.000	1.000	1.000	1.000	0.346	1.000	1.000	0.030	0.000						
EGR_3	1.000	1.000	1.000	1.000	1.000	1.000	1.000	1.000	1.000	1.000	1.000	1.000	1.000	1.000	1.000	1.000	1.000	1.000	1.000	1.000	1.000	1.000	0.000	0.000					
CD21L	1.000	1.000	1.000	1.000	1.000	1.000	1.000	1.000	1.000	1.000	1.000	1.000	1.000	1.000	1.000	1.000	1.000	1.000	1.000	1.000	1.000	1.000	1.000	1.000	0.000				
ECP	1.000	1.000	1.000	1.000	1.000	1.000	1.000	1.000	1.000	1.000	1.000	1.000	1.000	1.000	1.000	1.000	1.000	1.000	1.000	0.696	1.000	1.000	1.000	1.000	1.000	0.000			
IL5_P	1.000	1.000	1.000	1.000	1.000	1.000	1.000	1.000	1.000	1.000	0.699	0.826	1.000	1.000	1.000	1.000	1.000	1.000	1.000	1.000	1.000	1.000	1.000	1.000	1.000	1.000	0.000		
SAE_IGE	1.000	1.000	0.869	0.366	1.000	1.000	1.000	1.000	1.000	1.000	1.000	1.000	1.000	1.000	1.000	1.000	1.000	1.000	1.000	1.000	1.000	1.000	1.000	1.000	1.000	1.000	0.503	0.000	
IGE_P	0.973	1.000	0.132	0.556	0.674	1.000	1.000	0.254	1.000	0.540	0.000	0.305	0.061	1.000	1.000	1.000	1.000	1.000	1.000	1.000	1.000	1.000	1.000	1.000	1.000	1.000	0.030	0.027	0.000
