# Supplementary material for: Activation-Induced Cytidine Deaminase (AID)-Associated Multigene Signature to Assess Impact of AID in Etiology of Diseases with Inflammatory Component
Source: PLoS One. 2011 Oct 3;6(10):e25611. doi: 10.1371/journal.pone.0025611 (PMC3184987; doi:10.1371/journal.pone.0025611)
Supplement: Text S1 — Results. (DOC) [file pone.0025611.s011.doc]

**Text S1**

###### *Results*

## Expression profiling of AID in normal tissues assayed by real-time PCR

Despite the increasing interest in characterizing of AID expression in disease-relevant specimens, limited information is available about AID expression in normal human tissues. We designed primer pairs to detect AID mRNA of mouse and human origin and performed expression profiling in normal tissues (multiple tissue panels, Clontech). In mouse tissues, the highest expression levels were detected in salivary gland followed by spleen, lung, testis, and thymus (**Figure S1, A**); data are generally in line with previously published RT-PCR- and Northern blot-based results [1]. In a panel of human tissues (**Figure S1, B**), the highest levels of expression were detected in thymus followed by thyroid gland, uterus, trachea, skeletal muscle, and salivary gland; AID was detected in fetal brain, but it was not expressed in adult brain tissue. Expression profiling data of normal human tissue panel indicate that AID mRNA can be detected in non-lymphatic tissues.

###### Reference List

###### 1. Muramatsu M, Sankaranand VS, Anant S, Sugai M, Kinoshita K, Davidson NO, Honjo T (1999) Specific Expression of Activation-induced Cytidine Deaminase (AID), a Novel Member of the RNA-editing Deaminase Family in Germinal Center B Cells. J Biol Chem 274: 18470-18476.
